# Carbohydrate Antigen 125 as a Marker of Decongestion and Early Outcomes in Acute Decompensated Heart Failure: Insights From a Tertiary Hospital in Western Kenya

**DOI:** 10.1155/crp/7099401

**Published:** 2026-07-25

**Authors:** Rebeccah Ayoti Abutika, Felix Ayub Barasa, David Lagat, Gideon Titus Ng’eno, Gerald S. Bloomfield

**Affiliations:** ^1^ Department of Cardiology, Moi University School of Medicine, Eldoret, Kenya, mu.ac.ke; ^2^ Department of Cardiology, Moi Teaching and Referral Hospital, Eldoret, Kenya, mu.ac.ke; ^3^ Department of Medicine, Division of Cardiology, Duke University, Durham, North Carolina, USA, unc.edu

**Keywords:** acute decompensated heart failure (ADHF), carbohydrate antigen 125 (CA125), clinical congestion score (CCS)

## Abstract

**Background:**

Acute decompensated heart failure (ADHF) is a major cause of morbidity and mortality, with congestion being the primary reason for hospitalization. Despite treatment, many patients are discharged with residual congestion, hence the growing interest in utilizing biomarkers as objective tools to guide decongestion therapy. Carbohydrate Antigen 125 (CA125) has emerged as a promising biomarker of systemic congestion, with potential advantages over routine markers. This study evaluated the relationship between CA125 changes and clinical congestion score (CCS) at discharge and assessed its association with 30‐day outcomes.

**Methods:**

This was a prospective cohort study conducted over seven months at Moi Teaching and Referral Hospital, a tertiary hospital in Western Kenya. Patients with ADHF were enrolled. CA125 levels were measured at admission and discharge using an automated immunoassay. In addition, other markers of congestion, i.e., CCS, NT‐proBNP levels, body weight, and inferior vena cava (IVC) diameter, were assessed at both admission and discharge. The 30‐day outcomes, which included all‐cause mortality and heart failure (HF) rehospitalizations, were recorded. Statistical analyses included correlation and generalized linear models.

**Results:**

A total of 213 patients were included, with an in‐hospital mortality rate of 23.5%. There was a significant correlation between changes in CA125 levels and improvements in CCS at discharge (*p* = 0.018). Patients with in‐hospital CA125 reductions had lower rates of the composite 30‐day outcome (all‐cause mortality or HF rehospitalization) at 15%, compared to 40% in those with rising CA125 levels.

**Conclusion:**

A reduction in CA125 levels during hospitalization is associated with better clinical decongestion and improved short‐term outcomes.

## 1. Background

Acute decompensated heart failure (ADHF) is a major cause of morbidity and mortality worldwide and remains a leading indication for hospitalization among patients with cardiovascular disease [1, 2]. In sub‐Saharan Africa, ADHF contributes substantially to the growing burden of cardiovascular disease, with reported in‐hospital mortality rates ranging from 8.7% to 28.4% [3]. In Kenya, ADHF accounts for approximately 3%–6% of hospital admissions, with mortality rates as high as 20%–30% [4–6].

Congestion in heart failure (HF) is a complex and dynamic process that reflects not only fluid accumulation but also its heterogeneous distribution across different compartments [7]. It can be conceptualized in terms of regional involvement—pulmonary (left sided) and systemic (right sided)—as well as fluid compartments, including intravascular, interstitial, and third‐space accumulation. Intravascular congestion, characterized by elevated filling pressures, may precede overt clinical signs and progress to tissue and serosal fluid accumulation, manifesting as pulmonary edema, peripheral edema, ascites, and pleural or pericardial effusions [7]. Importantly, these forms of congestion frequently coexist, and most patients exhibit overlapping features rather than discrete phenotypes.

Despite advances in treatment, congestion remains the predominant reason for hospitalization in ADHF patients and a major therapeutic target [8]. However, studies reveal that many patients are discharged with residual congestion, and this is associated with higher rates of mortality and rehospitalization [9, 10]. This highlights the need for better strategies to monitor and manage fluid overload in hospitalized ADHF patients. While invasive hemodynamic monitoring, such as pulmonary artery catheterization, provides accurate measurements of congestion, its routine use is limited due to its invasive nature and lack of demonstrated benefit in improving outcomes [11].

Noninvasive clinical examinations largely rely on subjective evaluations, which can lead to variability between healthcare providers [12]. Composite clinical scores to evaluate congestion more accurately have been used in several trials in HF [13]. This approach seeks to address the limitations of individual signs and symptoms, which often lack sensitivity and specificity when used in isolation. The clinical congestion score (CCS) derived from a post hoc analysis of the Efficacy of Vasopressin Antagonism in Heart Failure Outcome Study with Tolvaptan (EVEREST) trial, is a composite congestion score calculated for patients hospitalized for worsening HF by adding the individual scores for each of the following (assessed on a standardized four point scale ranging from 0 to 3): leg edema, dyspnea, orthopnea, JVD, fatigue, and rales. The discharge value of this score was shown to significantly correlate with poor outcomes, i.e., mortality and recurrent HF hospitalization [8].

There is currently a growing interest in utilizing biomarkers as objective tools to assess congestion and guide decongestion therapy. While NT‐proBNP remains a widely used biomarker, its limitations have driven the search for alternatives. Carbohydrate Antigen 125 (CA125), encoded by the MUC16 gene, plays a significant role in the pathophysiology of HF, particularly in relation to systemic congestion. Elevated levels of CA125 in patients with HF are hypothesized to result from increased hydrostatic pressure, mechanical stress, and inflammatory stimuli that activate mesothelial cells on serosal surfaces (such as pleura, peritoneum, and pericardium). These stimuli lead to the production and release of CA125 into circulation [14]. However, CA125 is not specific to HF and may also be elevated in a range of noncardiac conditions, including ovarian and other malignancies, serosal inflammation (such as pleural, peritoneal, or pericardial disease), liver cirrhosis with ascites, endometriosis, and postoperative states.

In acute heart failure (AHF), higher CA125 levels are positively correlated with tissue congestion, pleural effusion, and elevated cardiac filling pressures, which are key markers of right‐sided HF. Studies, including one involving 2949 patients hospitalized for AHF, have linked CA125 levels with clinical factors such as tricuspid regurgitation severity, peripheral edema, and serous effusions [15]. This highlights the biomarker’s utility in reflecting systemic congestion and disease severity.

Unlike NT‐proBNP, CA125 is less affected by factors such as age, renal function, and weight, making it a potentially more reliable marker for decongestion. Research has also shown that CA125 levels correlate with other congestion markers, such as bioadrenomedullin and NT‐proBNP, and may even outperform NT‐proBNP in predicting mortality in patients with systemic congestion and right ventricular failure [15, 16]. Additionally, CA125 offers several advantages, including affordability, widespread availability, and high reproducibility, as it can be measured using standardized techniques and is unaffected by common preanalytical variables [17, 18]. These characteristics make CA125 an attractive biomarker for routine use in clinical practice, especially in resource‐constrained settings like Kenya.

Although the role of CA125 as a therapeutic biomarker to guide decongestion is increasingly being explored in high‐income countries, its application remains largely underexplored in other regions, including sub‐Saharan Africa. Addressing this knowledge gap is important given the differing etiological profiles of HF across regions. This is particularly relevant in Kenya, where the etiological profile of HF differs from that of high‐income countries, with a higher prevalence of valvular heart disease and nonischemic cardiomyopathies [5, 6]. Understanding the role of CA125 in this context could provide valuable insights into its clinical applicability and help bridge the knowledge gap for African populations.

Therefore, the main objectives of this study were to determine the correlation between changes in CA125 levels and CCSs at discharge and assess its association with 30‐day mortality and rehospitalization rates.

## 2. Methodology

### 2.1. Study Setting and Target Population

This was a single‐center prospective observational study conducted at Moi Teaching and Referral Hospital (MTRH), a tertiary referral hospital in Western Kenya. A total of 213 patients with ADHF were enrolled over a seven‐month period from December 2023 to June 2024. Ethical approval was obtained from the MTRH and Moi University Institutional Research and Ethics Committee (Ref: IREC/615/2023; Approval No. 0004589).

Eligible participants were identified through daily screening of admissions with a primary diagnosis of ADHF or presenting with dyspnea. ADHF was defined based on the presence of typical signs and symptoms of HF, together with objective evidence of structural and/or functional cardiac abnormalities on echocardiography—including reduced or preserved left ventricular ejection fraction, ventricular hypertrophy, chamber dilatation, significant valvular heart disease, or diastolic dysfunction—along with an NT‐proBNP level > 300 pg/mL, in accordance with the 2021 European Society of Cardiology guidelines. Patients were required to have a CCS ≥ 3 at admission. Both de novo and previously diagnosed HF patients were eligible for inclusion.

Patients aged ≥ 13 years were included. Written informed consent was obtained from all participants, with assent obtained from minors and consent from their legal guardians. Patients with known or suspected malignancy, a prior history of malignancy, or pregnancy were excluded.

## 3. Data Collection

Data were collected using a semistructured questionnaire capturing sociodemographic characteristics, comorbidities, and clinical parameters. Congestion was assessed using the CCS, which was calculated at admission and discharge. CCS is a composite score ranging from 0 to 18, derived from six clinical variables: dyspnea, orthopnea, fatigue, pulmonary rales, jugular venous pressure, and peripheral edema. Each component was graded on a standardized four‐point scale from 0 (*absent*) to 3 (*severe*), with higher scores indicating greater congestion (Table 1). The assessment was performed by the treating physician or a trained research assistant using a standardized protocol. Successful decongestion was defined as a CCS of 0 at discharge.

**TABLE 1:** Clinical congestion score.

Signs/symptoms	0	1	2	3
Dyspnea at rest	None	Seldom	Frequent	Continuous
Orthopnea	None	Seldom	Moderate	Continuous
Fatigue	None	Seldom	Frequent	Continuous
Basal crackles	None	Bases	To < 50%	To > 50%
Peripheral edema	None	Mild (clear pitting)	Moderate (above ankle)	Marked (above knee)
Jugular venous pressure (cm H_2_0)	< 6	6–9	10–15	> 15

Blood samples for CA125 and NT‐proBNP were obtained at two time points: at admission (T1) and at discharge (T2). Admission samples were collected within 24 h of presentation, prior to or early after initiation of decongestive therapy, while discharge samples were obtained within 24 h before hospital discharge. Approximately 2 mL of venous blood was collected and transported promptly to the MTRH biochemistry laboratory for analysis. Serum CA125 levels were measured using the SNIBE Maglumi 600 automated chemiluminescent immunoassay system (Shenzhen New Industries Biomedical Engineering Co., Ltd., China), while NT‐proBNP levels were measured using a standardized electrochemiluminescence immunoassay. All assays were performed according to manufacturer specifications.

Additional markers of congestion included body weight and inferior vena cava (IVC) diameter, both measured at admission and discharge. Weight was measured using a calibrated digital scale with patients in light clothing and without shoes. IVC diameter was assessed via transthoracic echocardiography using a subcostal long‐axis view, measured 1‐2 cm from the right atrial junction during the respiratory cycle. All measurements were performed by a trained sonographer and verified by a supervising cardiologist. Therapeutic management, including diuretic therapy, was administered at the discretion of treating physicians in accordance with standard clinical practice.

Adverse events, including mortality and HF rehospitalization, were key outcome measures in this study. Data on these events were obtained through a combination of direct follow‐up, medical record reviews, and telephone interviews conducted within 30 days postdischarge.

Mortality was assessed as all‐cause mortality due to limitations in reliable cause‐specific adjudication. Rehospitalization was defined as an unplanned hospital admission primarily due to worsening signs or symptoms of HF, necessitating either augmentation of oral HF medications or initiation of intravenous therapies, such as diuretics or inotropes.

Confirmation and adjudication of adverse events followed a standardized protocol. For mortality, the primary sources of information included hospital death records, official death certificates, or reliable accounts from immediate family members or caregivers. The principal investigator reviewed all death reports to confirm the accuracy of the cause of death.

Rehospitalizations were verified by reviewing hospital records to confirm the admission diagnosis, symptoms, and treatment interventions. A trained research assistant collected these data, which were then reviewed and adjudicated by the principal investigator. All events were carefully documented, and discrepancies, if any, were resolved through consultation with the study team to maintain consistency and reliability in the outcome assessment.

### 3.1. Data Analysis

Patients were categorized into quartiles based on the change in CA125 levels from admission to discharge as follows: patients in Q1 had a reduction in CA125 with discharge levels (T2) ≤ 35 U/mL; Q2 also showed a reduction, but T2 levels remained ≥ 35 U/mL; Q3 showed an increase in CA125 but with T2 levels ≤ 35 U/mL; and Q4 demonstrated both an increase in CA125 and T2 levels ≥ 35 U/mL. Baseline characteristics (both overall and among CA125 quartiles) were described using frequencies and percentages for categorical variables, while continuous variables were described using means with standard deviations or medians with interquartile ranges as appropriate.

Discharge CCSs were classified into three categories: a score of 0 indicated successful decongestion, a score of 1‐2 represented mild congestion, and a score > 3 indicated significant congestion. To analyze the relationship between changes in CA125 levels from admission to discharge and CCSs at discharge, we employed a generalized linear model adjusted for key baseline characteristics such as age, gender, duration of HF, weight, BUN, creatinine, albumin, and ALT to mitigate bias from regression to the mean in this change score analysis.

The association between changes in CA125 levels and a composite outcome of 30‐day all‐cause mortality and HF rehospitalizations was assessed by comparing the event rates among the CA125 quartiles. Pearson’s test was used to assess the correlation between changes in CA125 levels and other decongestion markers (weight, IVC diameter, and NT‐proBNP) with statistical significance also defined as *p* < 0.05.

## 4. Results

We studied a total of 213 patients who met the inclusion criteria. Median age was 61 years, 54.9% were female and 97% were hospitalized with NYHA class III/IV symptoms. Hypertension (46.9%) and atrial fibrillation (16.9%) were the most common comorbidities. The main causes of HF were mostly due to hypertensive heart disease (HHD) at 27.7%, followed by dilated cardiomyopathy (DCM) at 21.6%, right heart failure (RHF) at 20.2%, and rheumatic heart disease (RHD) at 19.7%. Approximately 43% of the patients had been hospitalized at least twice over the previous 12 months.

The median CCS at baseline was 9 (IQR 7–11), whereas the median CA125 and NT‐proBNP at baseline were 92.7 U/mL (IQR 38–163 u/mL) and 5639.5 pg/mL (IQR 2487 – 14,263.2 pg/mL), respectively. A total of 25% of the patients were on inotropic support. The in‐hospital mortality rate was 23.5%, and these patients had a markedly higher median CA125 level of 119.5 U/mL, compared to the overall cohort median of 92.7 U/mL. Baseline characteristics of the study cohort are presented in Table 2.

**TABLE 2:** Baseline characteristics of the study cohort (*N* = 213).

Characteristic	Value
Age (years)	Median: 61 (IQR: 42–76)
Gender	Female: 117 (54.9%)
Medical history	
Hypertension	100 (46.9%)
Diabetes	19 (8.9%)
Atrial fibrillation	36 (16.9%)
COPD	24 (11.3%)
Previous ACS	6 (2.8%)
Previous CVA	3 (1.4%)
Tobacco use	22 (10.3%)
RVD	5 (2.4%)
Heart failure etiology	
Hypertensive heart disease (HHD)	59 (27.7%)
Dilated cardiomyopathy (DCM)	46 (21.6%)
Right heart failure (RHF)	43 (20.2%)
Rheumatic heart disease (RHD)	42 (19.7%)
Ischemic heart disease (IHD)	9 (4.2%)
CHD	5 (2.4%)
Others	9 (4.2%)
HF Hospitalizations (previous 12 months)	
None	50 (23.5%)
1	70 (32.9%)
≥ 2	93 (43.7%)
SBP (mmHg)	Median: 109 (IQR: 93–128)
Weight (kg)	Median: 66.2
NYHA Class	
II	6 (2.8%)
III	85 (39.9%)
IV	122 (57.3%)
Laboratory parameters	
Hb (g/dL)	Median: 12.1 (IQR: 10–14)
Creatinine (umol/L)	Median: 90 (1QR 68–138)
NT‐proBNP (pg/mL)	Median: 5639.5 (IQR: 2487–14163.2)
CA125 (U/mL)	Median: 92.7 (IQR: 38–163)
Electrocardiogram findings	
Atrial fibrillation	73 (34.3%)
LBBB	14 (6.6%)
RBBB	4 (1.9%)
EF	
< 40%	92 (43%)
40%–49%	25 (12%)
> 50%	96 (45%)
Inotropic support	54 (25%)

### 4.1. Admission to Discharge Changes in Clinical and Biomarker Parameters

The median CCS decreased by seven points overall, with a greater reduction observed in Q1 (−8.0 points) compared to Q4 (−6.0 points, *p* = 0.018). Similarly, CA125 levels decreased significantly across the cohort, with the largest median reduction in Q1 (−157.4 U/mL) and an increase in Q4 (+36.2 U/mL, *p* < 0.001). NT‐proBNP levels showed median reductions from admission to discharge, but these were not significantly different among quartiles (*p* = 0.414). Other congestion‐related parameters such as weight and IVC diameter change, showed moderate reductions but were not statistically significant across quartiles (*p* = 0.177 and *p* = 0.717, respectively). Table 3 provides a summary of the admission to discharge difference in congestion parameters.

**TABLE 3:** Admission‐to‐discharge difference in congestion parameters.

	Total (*N* = 163)	Q1 (*N* = 41)	Q2 (*N* = 41)	Q3 (*N* = 40)	Q4 (*N* = 41)	*p* value
CCS change						
Median (IQR)	−7.0 (−10–−5.0)	−8.0 (−11–−7.0)	−7.0 (−10–−4)	−7.0 (−9–−5.5)	−6.0 (−8–−5.5)	0.018
Change in CA125 u/mL						
Median (IQR)	−16.7 (−76.3–5.3)	−157.4 (−215.4–−119.0)	−49.8 (−61.2–−32.0)	2.1 (0.7–6.6)	36.2 (21.0–106)	< 0.001
Change in NT‐proBNP pg/mL						
Median (IQR)	−1451.0 (−6630–−173.2)	−1770.0 (−7456–−466.2)	−2078.5 (−10592–−326.8)	−1191.0 (−4410.6–−600.8)	−1353.5 (−5924–−940.2)	0.414
Weight change kg						
Median (IQR)	−4.0 (−7–−2)	−4.0 (−8–−2.6)	−3.0 (−5.4–−1.0)	−4.0 (−7.3–−1.8)	−4.0 (‐6–−2.5)	0.177
Change in IVC diameter, cm						
Median (IQR)	−0.3 (−0.6–−0.1)	−0.5 (−0.5–−0.7)	−0.3 (−0.3–−0.5)	−0.2 (−0.2–−0.6)	−0.3 (−0.3–−0.6)	0.717

### 4.2. Correlation Between Changes in CA125 Levels and CCSs at Discharge

**FIGURE 1:**
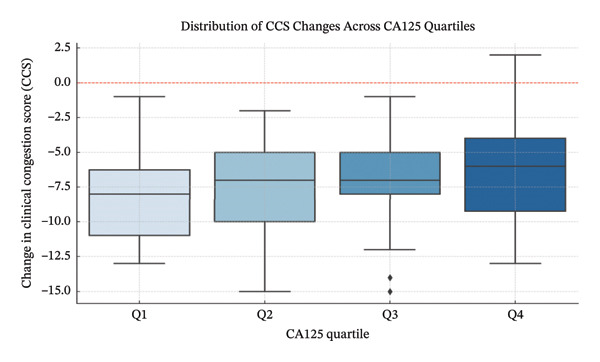
Distribution of CCS changes across CA125 quartiles.

Figure 1 illustrates the distribution of CCS changes across four CA125 quartiles. Patients in this Q1 had the largest median reduction in CCS (−8 points), indicating significant clinical decongestion, while those in Q2 and Q3 had a slightly lower median reduction in CCS (−7 points). Patients in Q4 had the least median CCS improvement (−6 points).

**FIGURE 2:**
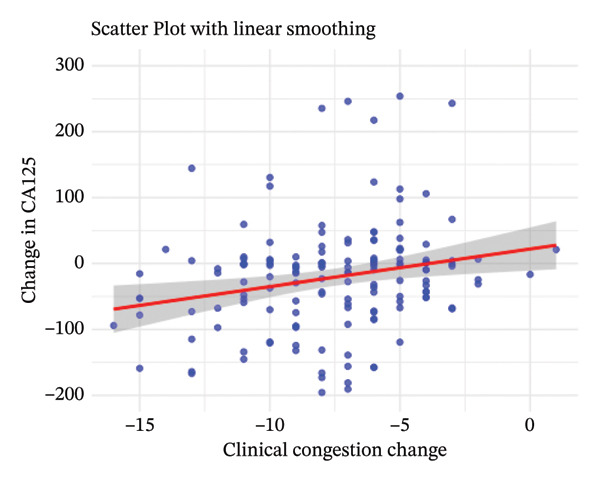
Scatter plot with linear smoothing.

The relationship between changes in clinical congestion and changes in CA125 levels was evaluated using a scatter plot with linear smoothing (Figure 2). A positive correlation was observed, as indicated by the fitted regression line (red) with a 95% confidence interval (shaded gray region). Specifically, as clinical congestion improved (i.e., more negative values for clinical congestion change), CA125 levels decreased.

The scatterplot, however, revealed substantial variability in CA125 changes, with values ranging from approximately −200 to +300. Despite this variability, the overall trend indicated that improvements in congestion were linked with decreases in CA125 levels.

### 4.3. Association Between Changes in CA125 Levels and 30‐Day Clinical Outcomes

Patients in Q1 had the lower rates of the composite 30‐day outcome (all‐cause mortality or HF rehospitalization) at 15% compared to 40% in Q4. Table 4 provides a summary.

**TABLE 4:** Association between CA125 quartiles at discharge and 30‐day clinical outcomes.

Outcome	Q1 (*n* = 41)	Q2 (*n* = 41)	Q3 (*n* = 40)	Q4 (*n* = 41)	*p* value
Mortality	0 (0%)	2 (4.9%)	1 (2.5%)	3 (7.3%)	0.331
Rehospitalization	6 (14.6%)	10 (24.4%)	12 (30%)	15 (33.6%)	0.141
Combined outcome	6 (14.6%)	12 (29.3%)	13 (33%)	18 (43.9%)	

### 4.4. Correlation Between Changes in CA125 and Other Markers of Decongestion

**FIGURE 3:**
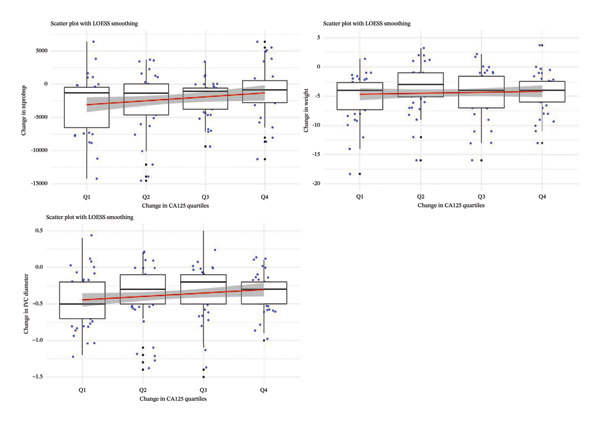
Correlation between changes in CA125 and other markers of decongestion.

A modest positive correlation was observed between CA125 changes and NT‐proBNP changes. Patients in Q1 and Q2, with decreasing CA125, generally showed greater reductions in NT‐proBNP levels. Conversely, Q4 patients exhibited smaller NT‐proBNP reductions. A weak positive correlation was seen between changes in CA125 and IVC diameter, with patients in Q1 and Q2 experiencing greater reductions in IVC diameter, while patients in Q4 showed minimal changes in IVC diameter. Conversely, the relationship between CA125 changes and weight loss was negligible, as evident from the nearly flat LOESS line across quartiles. Figure 3 provides a summary of the correlations.

## 5. Discussion

CA125, a high‐molecular‐weight glycoprotein produced by mesothelial cells, is increased in response to both serosal effusions and proinflammatory cytokine stimuli and has been found to correlate with clinical, echocardiographic, and hemodynamic markers of congestion [12]. Miñana et al. [15] highlighted the role of CA125 as a congestion biomarker by demonstrating its strong associations with pleural effusion, tricuspid regurgitation severity, and peripheral edema [13].

Our study supports these findings by showing that reductions in CA125 levels during hospitalization correlated significantly with improvements in CCSs at discharge. Patients in the lowest quartile of CA125 at discharge had the most pronounced improvement, while those in the highest quartile showed minimal clinical decongestion. These results further strengthen the hypothesis that CA125 is not merely a static reflection of baseline congestion but is a responsive biomarker capable of tracking decongestion in real time. This temporal association provides further support for its potential role in guiding therapy, particularly diuretic titration, as hypothesized by Núñez et al., who proposed a CA125‐guided therapeutic strategy in the CHANCE‐HF trial [17].

Patients with persistently elevated or rising CA125 levels at discharge had a numerically higher risk of 30‐day mortality and rehospitalization in our study cohort compared to those who achieved substantial reductions. This is in line with the results of Vader et al. [10], who demonstrated that residual clinical congestion at discharge was independently associated with poor 60‐day outcomes, including death, rehospitalization, and urgent medical visits. These findings reinforce the clinical value of CA125 not only as a dynamic biomarker of decongestion but also a biomarker that is capable of identifying patients at higher risk who may benefit from intensified follow‐up or therapeutic adjustment.

A notable insight from our study is the complementary link between CA125 and NT‐proBNP. While NT‐proBNP primarily reflects cardiac stress and left‐sided hemodynamic load [19], CA125 is more specifically linked to serosal and systemic manifestations of right‐sided congestion. This pathophysiological distinction likely resulted in the modest correlation between changes in CA125 and NT‐proBNP observed in our analysis, echoing prior reports [13]. As such, these two biomarkers appear to capture different yet synergistic aspects of HF physiology. The integration of both in clinical assessment could provide a more comprehensive evaluation of congestion and therapeutic response, a strategy previously advocated by Mullens et al. [20] in their proposed multimodal biomarker‐guided approach to decongestion [18].

In resource‐limited settings, CA125 offers several practical advantages over NT‐proBNP. It is less affected by confounding variables such as age, renal impairment, and body weight. Moreover, CA125 is cost‐effective and widely accessible, with well‐established measuring methods guaranteeing good reproducibility. These attributes render CA125 especially beneficial for implementation in healthcare systems with limited resources, such as those in sub‐Saharan Africa.

Interestingly, while traditional markers of decongestion, such as weight changes and IVC diameter, are commonly used, our study demonstrated negligible correlations between CA125 change with IVC diameter and weight. This may have been because weight changes can be confounded by factors such as diuretic resistance and nutritional status, while IVC diameter primarily reflects venous congestion rather than systemic fluid overload.

The findings of this study have important clinical implications, particularly for settings where advanced diagnostic tools are not readily available. By offering a simple, objective, and cost‐effective means of assessing congestion, CA125 could improve the accuracy of decongestive therapy and reduce the risk of residual congestion at discharge. This is especially relevant given the high rates of early readmission and mortality observed in ADHF patients with incomplete decongestion. Incorporating CA125 into routine practice could help clinicians identify high‐risk patients who may benefit from more aggressive or prolonged decongestive treatment.

CA125’s ability to predict short‐term outcomes could also provide an opportunity to tailor postdischarge management strategies. Patients with persistently elevated CA125 levels at discharge can be prioritized for closer follow‐up, including more frequent outpatient visits and earlier adjustments in their therapeutic regimens.

Despite the promising results, this study has several limitations. First, as a single‐center study, the findings may have limited generalizability to other populations and healthcare settings. Second, the cohort included a heterogeneous mix of HF etiologies, which may influence biomarker behavior and limit etiological‐specific interpretation. Third, congestion phenotypes were not formally differentiated, and the observed associations should, therefore, be interpreted as reflecting overall congestion rather than specific intravascular, pulmonary, or systemic domains. Fourth, although CA125 is a promising biomarker of congestion, it may be influenced by noncardiac conditions such as serosal inflammation, liver disease, or malignancy, which were not systematically excluded or adjusted for in this study.

Fifth, detailed therapeutic data, including cumulative diuretic dosing (expressed as furosemide equivalents), use of ultrafiltration, and invasive fluid removal procedures such as thoracentesis, were not systematically recorded, limiting the ability to directly link treatment intensity with biomarker changes. Similarly, CA125 levels were not measured in pleural or other serosal fluids. Finally, outcomes were limited to 30‐day follow‐up, and HF‐specific mortality could not be reliably distinguished from all‐cause mortality.

Subsequent research should seek to corroborate these results in larger, multicenter populations and investigate the efficacy of CA125 in conjunction with other novel biomarkers. Longitudinal studies examining the long‐term prognostic significance of CA125 and its utility in guiding outpatient care of HF are necessary. Standardized cutoff values for CA125 across various clinical settings should be created to promote its extensive integration into clinical practice [21–24].

## 6. Conclusion

Among patients admitted with ADHF at a tertiary hospital in western Kenya, reduction in CA125 was associated with improvement in clinical decongestion and reduction in 30‐day all‐cause mortality and hospitalization.

During the preparation of this work, the authors minimally used ChatGPT in order to improve language. After using this tool/service, the authors reviewed and edited the content as needed and take full responsibility for the content of the publication.

## Funding

This study was funded by Duke Global Health Institute, Duke University, 10.13039/100006511, GR000614.

## Conflicts of Interest

The authors declare no conflicts of interest.

## Data Availability

The data that support the findings of this study are available from the corresponding author upon reasonable request.
